# The effect of metformin on breast cancer outcomes in patients with type 2 diabetes

**DOI:** 10.1002/cam4.259

**Published:** 2014-06-18

**Authors:** Bridget A Oppong, Lindsay A Pharmer, Sabine Oskar, Anne Eaton, Michelle Stempel, Sujata Patil, Tari A King

**Affiliations:** 1 Breast Service, Department of Surgery, Memorial Sloan Kettering Cancer CenterNew York, New York, 10065; 2 Department of Epidemiology and Biostatistics, Memorial Sloan Kettering Cancer CenterNew York, New York, 10065

**Keywords:** Breast neoplasms, diabetes mellitus, type 2, metformin, mortality

## Abstract

Observational data suggest that metformin use decreases breast cancer (BC) incidence in women with diabetes; the impact of metformin on BC outcomes in this population is less clear. The purpose of this analysis was to explore whether metformin use influences BC outcomes in women with type 2 diabetes. Prospective institutional databases were reviewed to identify patients with diabetes who received chemotherapy for stages I–III BC from 2000 to 2005. Patients diagnosed with diabetes before or within 6 months of BC diagnosis were included. Males and those with type I, gestational, or steroid-induced diabetes were excluded. Patients were stratified based on metformin use, at baseline, defined as use at time of BC diagnosis or at diabetes diagnosis if within 6 months of BC diagnosis. Kaplan–Meier methods were used to estimate rates of recurrence-free survival (RFS), overall survival (OS), and contralateral breast cancer (CBC). We identified 313 patients with diabetes who received chemotherapy for BC, 141 (45%) fulfilled inclusion criteria and 76 (54%) used metformin at baseline. There were no differences in clinical presentation or tumor characteristics between metformin users and nonusers. At a median follow-up of 87 months (range, 6.9–140.4 months), there was no difference in RFS (*P* = 0.61), OS (*P* = 0.462), or CBC (*P* = 0.156) based on metformin use. Five-year RFS was 90.4% (95% CI, 84–97) in metformin users and 85.4% (95% CI, 78–94) in nonusers. In this cohort of patients with type 2 diabetes receiving systemic chemotherapy for invasive BC, the use of metformin was not associated with improved outcomes.

## Introduction

Diabetes mellitus (DM) affects 25.8 million people in the United States; 8.3% of the population 1. It is estimated that 8–18% of cancer patients have DM 2. Patients with DM are not only at higher risk for breast cancer (BC) 3,4 but DM is also believed to worsen BC prognosis 5–8. Metformin, a biguanide agent, is used as first-line therapy for the treatment of type 2 diabetes, particularly in overweight and obese patients. Emerging laboratory data suggest that this ubiquitous drug has anticancer potential, and observational studies suggest that metformin use decreases overall BC incidence among diabetics 9–12. While limited, these data have intensified the investigation of metformin and its potential role in BC prevention 13. At present there are multiple ongoing clinical trials attempting to address the clinical and biological role of metformin in BC.

There are several potential mechanisms by which metformin may impact breast carcinogenesis. In laboratory models, metformin stimulates AMP-activated protein kinase activity, thus inhibiting the mammalian target of rapamycin (mTOR) and decreasing proliferation in BC cell lines 14–16. Other potentially protective actions of metformin include inhibition of phosphorylation of IGF-1R/IR 17, inhibition of aromatase expression 18, and reduction in HER2 expression and its tyrosine kinase activity 14,19. When used in the management of diabetes, metformin reduces hyperinsulinemia and hyperglycemia, and improves insulin resistance 20, both of which are factors linking diabetes and cancer 21,22.

Although emerging data would imply the choice of antidiabetic pharmacotherapy might influence the course of BC, the clinical impact remains uncertain. Specifically, whether there is a survival advantage for women newly diagnosed with BC taking metformin is unknown. The study objective was to determine the impact of metformin use on BC outcomes in diabetic women receiving chemotherapy.

## Material and Methods

### Patients

Prospective institutional databases were retrospectively reviewed to identify patients who reported a diagnosis of DM and received systemic chemotherapy for stages I–III BC from 1 January 2000 through 31 December 2005 at Memorial Sloan Kettering Cancer Center (MSKCC). Male patients, those with type 1 DM, gestational, or steroid-induced diabetes, and those diagnosed with DM greater than 6 months after BC diagnosis were excluded. This study was approved by the Institutional Review Board.

Patients were stratified based on metformin use at baseline, defined as use at time of BC diagnosis or at time of diabetes diagnosis if that occurred within 6 months of BC diagnosis. Patient and tumor characteristics, glycemic control agents, cancer treatments, and subsequent BC events were obtained from the medical record and summarized for metformin users and nonusers. Differences were tested using the Wilcoxon rank-sum test for continuous variables and Fisher's exact test for categorical variables.

Cancer events occurring after diagnosis were classified as local recurrence, regional recurrence, contralateral breast cancer (CBC), or distant metastases. Local recurrence was defined as histologic proven tumor reappearance in the ipsilateral breast or chest wall. Recurrence in the internal mammary, supraclavicular, or ipsilateral axillary nodes was classified as regional recurrence. All other sites of tumor recurrence were classified as distant metastases. CBCs occurring within 6 months of the index cancer diagnosis were considered synchronous diagnoses; later events were considered CBC.

Time-to-event variables were measured from the date of index surgery, using earliest surgery in cases of bilateral cancer, and censored at the end of follow-up. Recurrence-free survival (RFS) was defined as time until recurrence (local, regional, or distant), or death if the patient died without recurrence. Kaplan–Meier curves were generated for RFS, overall survival (OS), and CBC, stratified on metformin use, and curves were compared using the log-rank test. Women with bilateral cancer at diagnosis and women having contralateral prophylactic mastectomy were not included in the analysis of CBC rates. Cox models were used to assess the effects of stage, age, menopausal status, body mass index (BMI), lymph node positivity, estrogen receptor (ER) status, histologic grade, lymphovascular invasion, and years since diabetes diagnosis on OS and RFS. The effect of metformin on OS and RFS was further assessed in multivariable Cox models after adjusting for age, hormone receptor status, and stage.

Competing risks methods were used to estimate the cumulative incidence of local, regional, and distant recurrence. The first event among local, regional, and distant recurrence and death was counted as the primary event, and any ensuing events were censored at the date of the primary event. Gray's test was used to compare event rates between the groups in the case of competing risks. All statistical analysis was performed in SAS 9.2 (SAS Institute, Cary, NC) and R 2.11.1 (R Development Core Team, Vienna, Austria), and *P*-values less than 0.05 were considered significant.

## Results

From 2000 to 2005, 2889 patients received chemotherapy for primary stages I–III BC at MSKCC. Of these, 313 (9.2%) patients were documented as having diabetes and 141 (5%) fulfilled study inclusion criteria; 104 patients had type 2 diabetes at BC diagnosis and 37 were diagnosed with DM within 6 months of cancer diagnosis.

### Patient demographics and clinical characteristics

Clinical characteristics of the study population are shown in Table1. The majority of BC patients with DM were postmenopausal 120 (85%) with a median age of 61 years (range, 38–80 years). Median BMI was 32 kg/m^2^ (range, 17–55 kg/m^2^). There was no difference between metformin users and nonusers with regard to the timing of diabetes diagnosis; 57(92%) of metformin users and 47(89%) of nonusers were diagnosed with DM prior to their BC diagnosis. The median duration of time for which patients were known to be diabetic prior to BC diagnosis was 5.6 years and 5.3 years in the metformin and no-metformin groups, respectively. Among patients using metformin at baseline, the median duration of metformin use during follow-up, from date of BC diagnosis, was 5.9 years (range, 30 days–11.7 years). In the no-metformin group, 21 (32.3%) patients managed their diabetes with diet alone. There was no difference in the use of other oral hypoglycemics (*P* = 0.496) or insulin (*P* = 0.659) between the two groups.

**Table 1:** Study population characteristics.

Variable	No-metformin group (*n* = 65 patients)	Metformin group (*n* = 76 patients)	*P*-value
Age at BC diagnosis, years			
Median (range)	61 (42–80)	59 (38–75)	0.163
Race			0.891
Caucasian	31 (47.7%)	41 (54.7%)	
African American	25 (38.5%)	27 (36.0%)	
Asian	6 (9.2%)	4 (5.3%)	
Hispanic	2 (3.1%)	2 (2.7%)	
Data not available	0	1	
BMI, kg/m^2^			
Median (range)	30 (20–55)	33 (17–53)	0.316
Postmenopausal	59 (90.8%)	61 (80.3%)	0.099
Prior history of BC	5 (7.7%)	3 (3.9%)	0.471
Timing of DM diagnosis			0.752
Prior to BC diagnosis	47 (88.7%)	57 (91.9%)	
Within 6 months of BC diagnosis	6 (11.3%)	5 (8.1%)	
Missing	12	14	
Duration of DM prior to BC, years			
Median (range)^1^	5.3 (0.3–29.0)	5.6 (0.4–30.5)	0.979
DM medical management			
Diet control	21 (32.3%)	—	
Other oral DM medication^2^	34 (52.3%)	45 (59.2%)	0.496
Insulin use	13 (20.0%)	12 (15.8%)	0.659
Duration of metformin use, days			
Median (range)^3^	—	2167 (30–4288)	NA

BC, breast cancer; BMI, body mass index; DM, diabetes mellitus.

1 Among women who were diagnosed with DM prior to BC diagnosis and had date of DM diagnosis available. (Date of DM diagnosis was missing for 26 patients).

2 Other DM therapies included other oral hypoglycemic agents (sulfonylureas and thioazolidenediones).

3 Among women taking metformin at time of BC diagnosis, starting from date of BC diagnosis.

Index tumor characteristics are summarized in Table2. Two women in each group had bilateral BC resulting in the inclusion of 145 tumors in this analysis. There were no differences in clinical stage at diagnosis, final pathologic stage, or any other histologic variables between the two groups. The majority of patients had stage II or III ER-positive invasive ductal cancer. There were no differences in the rate of breast conservation versus mastectomy or adjuvant radiation between metformin users and nonusers. In the small subgroup of patients treated with neoadjuvant chemotherapy (*n* = 11), one patient achieved a complete pathologic response; the patient was in the metformin group.

**Table 2:** Study population: tumor characteristics and treatment.

Variable	No-metformin group (*n* = 67 tumors)	Metformin group (*n* = 78 tumors)	*P*-value
Tumor size, cm			
Median (range)	1.8 (0–6.8)	1.7 (0–6.0)	0.838
Histologic type			0.197
Ductal	51 (76.1%)	68 (87.2%)	
Lobular	8 (11.9%)	6 (7.7%)	
Other	8 (11.9%)	4 (5.1%)	
Histologic grade			0.178
1	3 (5.3%)	0 (0.0%)	
2	12 (21.1%)	15 (20.8%)	
3	42 (73.7%)	57 (79.2%)	
Data not available	10	6	
Lymphovascular invasion	26 (40.0%)	32 (41.0%)	1.000
Data not available	2	0	
Estrogen receptor			0.591
Positive	48 (71.6%)	52 (66.7%)	
Negative	19 (28.4%)	26 (33.3%)	
Progesterone receptor			0.240
Positive	33 (49.3%)	30 (38.5%)	
Negative	34 (50.7%)	48 (61.5%)	
HER2/neu			1.000
Positive	11 (16.7%)	13 (16.7%)	
Negative	55 (83.3%)	65 (83.3%)	
Data not available	1	0	
Pathologic stage			0.210
I	19 (28.4%)	14 (18.0%)	
II	31 (46.3%)	33 (42.3%)	
III	17 (25.4%)	29 (37.2%)	
IV	0 (0%)	1 (1.3%)	
pCR	0 (0%)	1 (1.3%)	
Surgery			1.000
Partial mastectomy	32 (48.5%)	38 (48.7%)	
Mastectomy	34 (51.5%)	40 (51.3%)	
Missing	1	0	
Postmastectomy radiation	16/34 (47.1%)	25/40 (62.5%)	0.242
Chemotherapy			0.224
Neoadjuvant	3 (4.5%)	8 (10.3%)	
Adjuvant	64 (95.5%)	70 (89.7%)	
Hormonal therapy (ER/PR+ tumors)	49 (100.0%)	54 (100.0%)	1.000
Trastuzumab (HER2+ tumors)	6 (54.5%)	5 (38.5%)	0.682

This cohort was largely treated before the standard use of trastuzumab in the adjuvant setting. pCR, pathologic complete response; ER, estrogen receptor; PR, progesterone receptor.

In a subset analysis of women who had DM prior to their BC diagnosis, we analyzed the presenting features of the cancers in metformin users versus nonusers. There were no differences in histologic subtype (ductal, lobular, other), pathologic stage, nodal status, histologic grade, presence of lymphovascular invasion, or hormone receptor status by metformin use (data not shown).

### Outcomes and survival estimates

**Figure 1:**
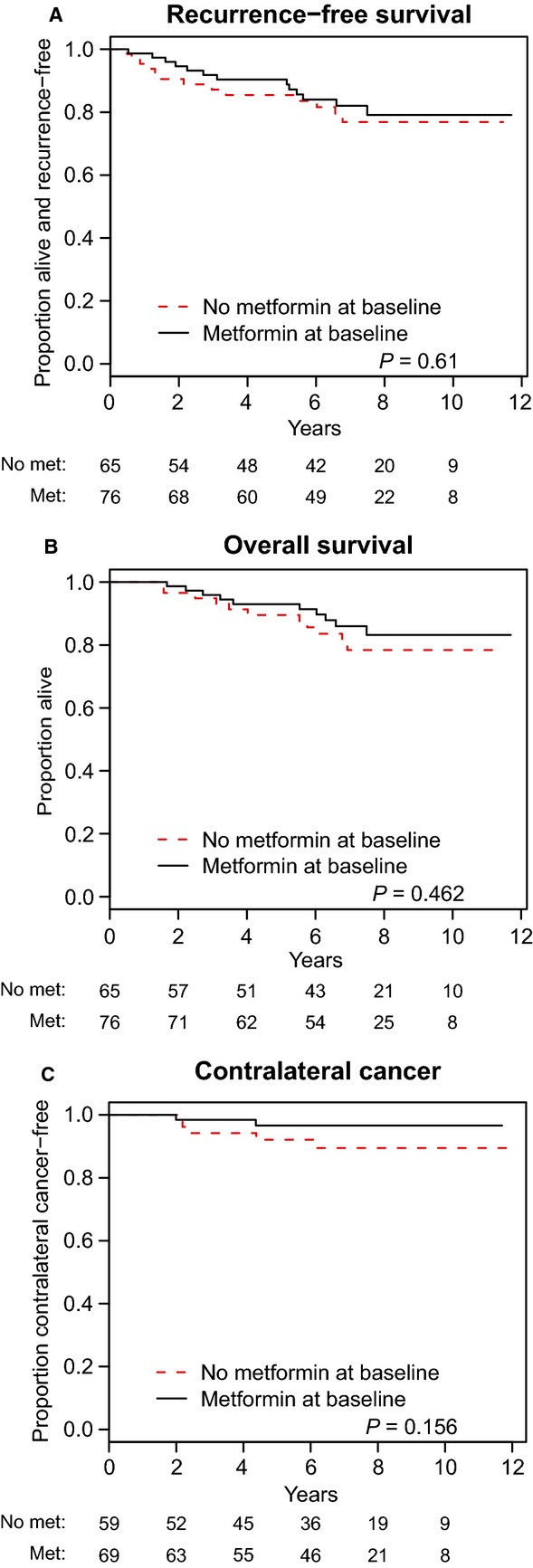
Kaplan–Meier estimates of (A) recurrence-free survival, (B) overall survival, and (C) contralateral breast cancer are shown stratified by study groups (metformin and no-metformin).

At a median follow-up of 87.0 months (range, 6.9–140.4 months), there was no difference in subsequent BC event rates or OS between the two groups (Table3). Kaplan–Meier curves for RFS (a composite of local, regional, and distant recurrence), OS, and CBC stratified by group (metformin vs. no-metformin) are shown in Figure1. At 5 years, RFS estimates were 90.4% (95% confidence interval [CI], 83.6–97.2) and 85.4% (95% CI, 76.6–94.3) in metformin users and nonusers, respectively (*P* = 0.610). Five-year OS estimates were 93.0% (95% CI, 87.0–98.9) and 89.7% (95% CI, 81.9–97.5) in metformin users and nonusers, respectively (*P* = 0.462), and CBC rates at 5 years were 3.4% (95% CI, 0–7.9) in the metformin group and 7.9% (95% CI, 0.1–15.1) in the no-metformin group (*P* = 0.156). Although crude rates of all events (local, regional, distant, and CBC) were lower in metformin users; these were not statistically significant. In univariate analysis, disease stage, age, menopausal status, BMI, lymph node positivity, ER status, histologic grade, lymphovascular invasion, and years since diabetes diagnosis were not associated with RFS or OS (all *P* > 0.05, results not shown). In multivariable analysis adjusting for select prognostic variables, metformin use was not associated with improved RFS or OS (Table4).

**Table 3:** Breast cancer outcomes.

	No-metformin group (*n* = 65 patients)	Metformin group (*n* = 76 patients)	*P*-value
Follow-up among survivors, months			NA
Median	88.0	86.4	
Range	10.6–137.4	6.9–140.4	
RFS at 5 years (95% CI)	85.4 (76.6–94.3)	90.4 (83.6–97.2)	0.610
OS at 5 years (95% CI)	89.7 (81.9–97.5)	93.0 (87.0–98.9)	0.462
CBC rate at 5 years (95% CI)	7.9 (0.1–15.1)	3.4 (0–7.9)	0.156
5-year event rates (95% CI)			
Local	3.1 (0–7.4)	0	0.126
Regional	1.5 (0–4.6)	0	0.917
Distant	9.9 (2.3–17.5)	8.2 (1.9–14.6)	0.876
Number of events			
OS	12	10	
RFS	13	13	
CBC	5	2	
Local recurrence	2	0	
Regional recurrence	1	1	
Distant recurrence	7	9	

NA, not available; RFS, recurrence-free survival; OS, overall survival; CBC, contralateral breast cancer; CI, confidence interval.

**Table 4:** Multivariable Cox models for recurrence-free survival and overall survival.

	HR (95% CI)	*P*-value
**Recurrence-free survival**		
Metformin use	0.86 (0.38–1.90)	0.70
Path stage		0.10
I	REF	
II	0.62 (0.22–1.80)	
III/IV	1.68 (0.62–4.55)	
Age	1.04 (1.00–1.09)	0.07
HR status		0.05
Negative	REF	
Positive	0.46 (0.21–1.01)	
**Overall survival**		
Metformin use	0.80 (0.33–1.96)	0.63
Age	1.06 (1.00–1.11)	0.03
Path stage		0.24
I	REF	
II	0.95 (0.29–3.17)	
III/IV	1.07 (0.62–6.89)	
HR status		0.02
Negative	REF	
Positive	0.35 (0.15–0.84)	

One patient with pCR excluded. CI, confidence interval; REF, reference; HR, hormone receptor.

## Discussion

Metformin is one of the most highly prescribed medications in the United States 23. In addition to its antidiabetic effects, observational data suggest that metformin may reduce BC risk and potentially alter BC survival. Here we present the results of a single-institution study of women with type 2 DM and invasive BC examining the effect of metformin use on BC outcomes. In this retrospective analysis of 141 women with DM who received chemotherapy for BC, the use of metformin did not result in significant improvements in RFS, OS, or CBC rates at a median follow-up of 7.3 years. It is noteworthy, however, that in this cohort of patients with primarily stage II and III BC requiring chemotherapy in the setting of comorbid disease, the 5-year event rates were considerably lower than historical series 24. This cohort was also largely treated before the standard use of trastuzumab in the adjuvant setting, and 37% of women in our study cohort self-reported as African American, both of which have been associated with poorer BC outcomes 25,26. Despite these factors, our 5-year RFS estimates (85.4–90.4%) were better than anticipated.

Prior studies examining the effect of metformin on BC mortality are reviewed in Table5. The studies from the United Kingdom and China are confounded in that they compare diabetics treated with metformin to nondiabetic patients, making it very difficult to draw conclusions 27,28, and the other studies generated inconsistent results. Bayraktar et al. 29 analyzed women who received adjuvant chemotherapy for triple-negative BC. After adjustment for age, body weight, tumor size, lymph node status, nuclear grade, lymphovascular invasion, and type of adjuvant chemotherapy received, they found no difference in distant metastasis-free survival, RFS, or OS between 63 diabetic women using metformin and 67 diabetic women not using metformin. In contrast, He et al. 30 found that among diabetic metformin users (*n* = 88) and nonusers (*n* = 66) with stage ≥2 HER2+ BC, metformin use was associated with decreased BC-specific mortality (*P* = 0.023; hazard ratio [HR], 0.47; 95% CI, 0.24–0.90). A recent Canadian population-based study of women ≥66 years of age with diabetes and BC failed to show a significant association between metformin therapy and all-cause mortality (adjusted HR, 0.97; 95% CI, 0.92–1.02) or BC-specific mortality (adjusted HR, 0.91; 95% CI, 0.81–1.03) using a cumulative time-varying exposure approach 31. However, a direct comparison with previous studies is difficult due to the lack of data on cancer stage and subtype.

**Table 5:** Reports of the effect of metformin on breast cancer mortality.

Study (1st author, country, year)	Design	Population	Comparison groups	*N* (per group)	Mean age (years)	Median/mean follow-up (years)	Outcome	Risk estimates (95% CI)	Adjusting variables
He X, USA, 2012	Retrospective cohort study	Type 2 diabetics with stage ≥2 HER2+ BC	Metformin users vs. nonusers	88 vs. 66	55	4	BC-specific mortality	HR = 0.47 (0.24–0.90), *P* = 0.023	Age, BMI, ER/PR status, insulin use
Bayraktar S, USA, 2012	Retrospective cohort study	TNBC with DM	Non-metformin users vs. metformin users	67 vs. 63	52	5.2	DMFS	HR = 1.63 (0.87–3.06)	Age, weight, tumor size, LN status, nuclear grade, LVI, type of adjuvant chemotherapy
							Recurrence-free survival	HR = 1.37 (0.78–2.40)	
Currie C, UK, 2012	Retrospective cohort study	BC	Type 2 diabetics vs. nondiabetics	1182 vs. 24,393	—	6.8	OS	DM vs. no DM HR = 1.32 (1.17–1.49)	Age, smoking, CCI, year of diagnosis
							OS	Metformin vs. no DM HR = 0.96 (0.68–1.37)	Age, smoking, CCI, year of diagnosis, Townsend index of deprivation, number of primary care contacts
Hou G, China, 2013	Retrospective cohort study	BC	Metformin users vs. non-metformin users vs. nondiabetics	419 vs. 594 vs. 4621	—	5.7	OS	Metformin vs. no DM HR = 0.76 (0.6–0.97) No metformin vs. no DM HR = 1.71 (1.46–2.0)	
Lega I, Canada, 2013	Population-based cohort study	Women 66 years of age with DM and BC	Metformin users vs non-metformin users	1094 vs. 1267	77.4	4.5 3.7	All-cause mortality BC-specific mortality	HR = 0.97 (0.92–1.02) HR = 0.91 (0.81–1.03)	Age, TZD, sulfonylurea, insulin use, duration of DM before BC, BC treatments, comorbidity score, DM medication prior to BC

CI, confidence interval; BC, breast cancer; HR, hazard ratio; BMI, body mass index; ER, estrogen receptor; PR, progesterone receptor; DM, diabetes mellitus; DMFS, distant metastasis-free survival; LN, lymph node; LVI, lymphovascular invasion; OS, overall survival; CCI, Charlson Comorbidity Index; TZD, thiazolidinediones; TNBC, triple negative breast cancer.

It has been proposed that the predominant mechanism of action of metformin may differ across BC molecular subtypes 32, yet at the cellular level, metformin has been shown to inhibit growth in trastuzumab-resistant HER2+ cells 33 and to inhibit cell proliferation and induce apoptosis in triple-negative BC cell lines 34, suggesting that the effect of metformin in the Bayraktar et al. 29 and He et al. 30 studies should have been similar. In our cohort, there were similar proportions of triple-negative cancers among metformin users (22%) and nonusers (20%), as well as the same percentage of HER2/neu-positive cancers (16.7%) in each group; however, these numbers were too small for subset analysis.

We did observe a lower rate of CBC in the group using metformin with a Kaplan–Meier estimated 5-year event rate of 7.9 in nonusers and 3.4 in users, suggesting a potential role for metformin in BC prevention, although not statistically significant in our small sample size. Data evaluating the relationship between diabetes and the risk of CBC are limited; however, in a population-based nested case–control study, Li et al. 35 compared 322 women with ER positive BC who developed CBC to 616 matched controls without CBC and demonstrated that diabetics had a 2.2-fold (95% CI, 1.3–3.6) increased risk of CBC. While data regarding the effect of metformin on BC incidence are inconsistent, with several studies showing a risk reduction 9–12 and others showing no benefit 8,36,37 or increased risk 38, a recent meta-analysis comparing metformin users to nonusers demonstrated a 6% risk reduction in the incidence of BC in metformin users 39. Therefore, it is plausible that diabetics have a baseline elevated risk of second primary breast malignancy, and if metformin is beneficial in reducing primary breast cancer risk, it may also prove to be beneficial in reducing the risk of CBC.

It is unclear whether metformin influences the development of specific histologic features or alters the clinical presentation of BC, although metformin use among diabetics has been associated with BCs that are less frequently triple negative 40. Overall, we did not observe a difference in any histopathologic features or hormone receptor status between users and nonusers of metformin. In the subset of women who had DM prior to their BC diagnosis, there were no differences in histologic subtype, pathologic stage, nodal status, histologic grade, presence of lymphovascular invasion, or hormone receptor status.

Jiralerspong et al. 41 found that diabetic patients with BC treated with metformin experienced higher pathologic response rates after neoadjuvant chemotherapy than those treated with other diabetic medications (24% vs. 8%; *P* = 0.007), although in their study, metformin was not found to be an independent predictor of either RFS or OS after adjusting for diabetic status, age, BMI, stage, grade, ER/progesterone receptor (PR) status, and neoadjuvant taxane use. Among our small subgroup of patients receiving neoadjuvant chemotherapy (*n* = 11), there was one patient who had a complete pathologic response; she was taking metformin. However, we found no association between metformin and either RFS or OS.

This study is limited by its retrospective nature and small sample size. In addition, our cohort consisted primarily of women who self-reported diabetes, with only a minority being diagnosed by an endocrinologist at our institution. Additionally, as a cancer center, we did not have access to patients' primary care medical records, which might contain more detailed information on glycemic control, including hemoglobin A1c and insulin levels. There are also concerns regarding confounding because metformin-treated patients may have different clinical characteristics than other diabetes-related treatment groups, such as those treated with diet alone, which we may not have been able to detect due to the small sample size. Yet when we limited our analysis to patients who developed BC after they were diagnosed with diabetes, we did not observe any significant differences in clinical presentation or features of the BCs that developed in metformin-treated patients versus those treated with diet alone or other agents.

Further research is needed to understand the potential effects of metformin therapy on BC. Metformin may have broad activity against carcinogenesis in general, making it a more attractive chemoprevention agent than disease site-specific compounds such as tamoxifen or raloxifene 17. This may lead to extended therapeutic uses for the drug. Our failure to show significant beneficial effect of metformin may represent the true absence of such an effect; or may reflect that our study was underpowered to detect these effects. Rates of CBC, for example, appeared to diverge between groups; however, it is likely that a much larger sample size would be necessary to detect a difference of the magnitude seen for CBC. In light of these limitations, we believe our study adds to the accumulating evidence justifying the investigation of the potential benefits of metformin on BC outcomes and prevention that are underway in many centers in Canada, Europe, and the United States. These include a large phase III trial led by the National Cancer Institute of Canada Clinical Trials Group (MA.32) to evaluate the effects of metformin in the adjuvant BC setting on survival outcomes, as well as over 20 clinical trials underway in the United States investigating metformin's role in BC.
